# Does numerical similarity alter age-related distractibility in working memory?

**DOI:** 10.1371/journal.pone.0222027

**Published:** 2019-09-04

**Authors:** Chiara Francesca Tagliabue, Debora Brignani, Veronica Mazza

**Affiliations:** 1 Center for Mind/Brain Sciences (CIMeC), University of Trento, Trento, Italy; 2 IRCSS Istituto Centro San Giovanni di Dio, Fatebenefratelli, Brescia, Italy; Zhejiang Univeristy, CHINA

## Abstract

Similarity between targets and distracters is a key factor in generating distractibility, and exerts a large detrimental effect on aging. The present EEG study tested the role of a new stimulus dimension in generating distractibility in visual Working Memory (vWM), namely numerical similarity. In a change detection paradigm a varying number of relevant and irrelevant stimuli were presented simultaneously in opposite hemifields. Behavioral results indicated that young participants outperformed older individuals; however, in both groups numerical similarity per se did not modulate performance. At the electrophysiological level, in young participants the Contralateral Delay Activity (CDA, a proxy for item maintenance in vWM) was modulated by the numerosity of the relevant items regardless of numerical similarity. In older participants, the CDA was modulated by target numerosity only in the same numerical condition, where the total number of (relevant and irrelevant) items increased with increasing target numerosities. No effect was present in the dissimilar numerical condition, where the total number of items did not vary substantially across target numerosity. This pattern was suggestive of an age-related effect of the total number of (relevant and irrelevant) items on vWM. The additional analyses on alpha-band lateralization measures support this interpretation by revealing that older adults lacked selective deployment of attentional and vWM resources towards the relevant hemifield. Overall, the results indicate that, while numerical similarity does not modulate distractibility, there is an age-related redistribution of vWM resources across the two visual fields, ultimately leading to a general decrease in task performance of older adults.

## Introduction

Feature similarity between targets and distracters is a key factor in generating distractibility during the execution of several tasks (e.g. [1, 2, 3]). For instance, it has been shown that when targets and distracters are similar in terms of primary physical properties (such as size, orientation, shape, color), they compete to enter the memory buffer [4]. Thus, highly similar distracters exert large distractibility, and thus worsen performance on the target items [5, 6].

The effect exerted by target-distracter similarity among physical features should have a large detrimental impact in aging. Aging is characterized by several physiological and functional modifications, among which deterioration of working memory (WM) is the most representative one [7]. According to several findings (e.g. [8, 9]), the age-related deterioration in visual WM (vWM) is due to an increase of distractibility, namely the inability to discard irrelevant information and focus only on the relevant objects [10], which in turn reduces the storage resources available in vWM. Recent EEG studies [11, 12, 13] addressing the neurophysiological substrates of the effect of aging on vWM indicated that the Contralateral Delay Activity (CDA; [14]), an electrophysiological index for vWM capacity, is indeed modulated by aging. This modulation has been interpreted as evidence of age-related differences in the efficiency to filter out irrelevant information from the vWM buffer, due to increase in distractibility for the elderly.

Results from studies on target-distracter similarity in aging [15, 16, 17, 18] are in line with this interpretation. For instance, older individuals are slower and less accurate than young participants when detecting targets embedded in conjunction-search displays with distracters highly similar for orientation and size [19]. Given these results, one should expect that similarity between targets and distracters exerts a detrimental effect in the healthy elderly population for all primary stimulus attributes.

Research in the past two decades has indicated object numerosity as a new stimulus attribute that is independent from other physical attributes, but can nonetheless be considered a primary visual property (e.g., see [20, 21, 22]; but see [23]). Thus, a straightforward prediction is that, as for the other primary attributes, *numerical* similarity between targets and distracters would impair performance during the execution of various tasks, and that the impairment would be larger in aging. To investigate this issue, the present study probed the contribution of target-distracter numerical similarity to distractibility in young and older adults performing a vWM task.

In a change detection task we presented a varying number of targets and distracters in the visual field. Crucially, their number was manipulated independently, in order to create conditions where targets and distracters shared the same numerosity (e.g. 2 targets and 2 distracters) and conditions of disparity between the two sets (e.g. 2 targets and 4 distracters; see also [24, 25]). From an ecological perspective, the manipulation of the similarity in the number of targets and distracters offers a good approximation to everyday scenarios. Indeed, in order to accomplish the majority of tasks (e.g. shopping at the supermarket), individuals typically deal with multiple relevant and irrelevant items that are presented simultaneously and with varying numerosities, rather than one isolated element against a constant number of distracters.

We predicted that in the same numerical condition, the redundant information due to target and distracter numerical similarity (e.g., the fact that there are two targets and two distracters) should induce inadvertent processing of the distracter elements. The additional processing of distracters should result in a reduction of the number of the consolidated target items with respect to the dissimilar numerical condition (where no numerical redundancy is present).

In terms of behavioral measures, we thus predicted a lower performance for the same distracter numerosity condition, compared to the dissimilar condition. Moreover, we expected the detrimental effect induced by numerical similarity (if present) to be larger in the older group, due to age-related increased distractibility [10].

In terms of EEG measures, our main focus was on the CDA and its modulation as a function of target numerosity for the same versus dissimilar distracter numerical conditions. In young adults, we expected a reduced modulation of the CDA amplitude as a function of target numerosity for the same numerical condition. As previously mentioned, distractibility is more evident in aging, as evidenced by a lack of suppression of the neural activity related to the processing of irrelevant material [8, 9] and its subsequent memorization [11, 12]. Given this greater age-related distractibility, the effect of numerical similarity (if present) should be larger in older than young participants. Thus, we expected a larger *reduction* of the CDA modulation as a function of target numerosity in the same versus dissimilar distracter numerical condition for older compared to young adults.

Finally, lateralization in alpha power is also measured during the retention interval in WM tasks [26], and it has been interpreted as evidence of suppression of irrelevant items. As contrasting evidence of aging effects on alpha lateralization has also been found in this time window (preserved: [27]; reduced: [28]), we additionally investigated the impact of distracter numerical similarity and aging on modulations in the alpha band activity after the memory array presentation.

## Materials and methods

### Participants

Thirty-three healthy young adults and 33 healthy older adults participated in the study. All reported normal or corrected-to-normal vision and a negative history of neurological or psychiatric disorders. Data from 2 young and 1 older participant were not included in the analyses due to excessive noise during EEG recording, resulting in a final sample of 31 younger adults (16 women; age range: 19–31; mean age ± standard deviation = 23.5 ± 3.3; mean education ± standard deviation = 15.7 years ± 1.8) and 32 older adults (16 women; age range = 63–79; mean age ± standard deviation = 69.8 ± 4.6; mean education ± standard deviation = 13 years ± 2.4). Written informed consent to participate in the study was obtained prior to testing. The study was approved by the Ethics Committee of the University of Trento and conducted in accordance with the 2013 Declaration of Helsinki.

### Neuropsychological testing

Older adults were administered a battery of neuropsychological tests in order to assess their cognitive fitness. The exclusion criterion was set to more than one test score below the cut-off values. None of the older participants was excluded on the basis of this criterion. The results for each cognitive test are shown in Table 1.

**Table 1:** Neuropsychological tests.

Neuropsychological Test	Mean Raw Score (SD)	Mean Correct Score (SD)	Cutoff
**MMSE** [29]	28.6 (1.5)	28.2 (1.7)	≤ 23.80
**RAVLT Immediate Recall** [30]	49.1 (9.9)	52.1 (9.5)	≤ 28.52
**RAVLT Delayed Recall** [30]	10.9 (3.6)	12 (3.5)	≤ 4.68
**Digit Span Forward** [31]	5.7 (0.9)	5.8 (0.9)	< 4.26
**Digit Span Backward** [31]	4.5 (1.1)	4.6 (1)	< 2.65
**RCPM 47** [32]	32.6 (3.4)	33.7 (3.2)	≤ 18
**Attentive Matrices** [33]	55.5 (3.2)	53.9 (4.1)	≤ 30
**TMT A** [34]	39.1 (10.9)	24.4 (11.3)	> 93
**TMT B** [34]	89.5 (22.4)	42.9 (23.9)	> 282
**TMT B-A** [34]	46.8 (18.4)	18.9 (19.9)	> 186
**ROCF Copy** [35]	32.6 (2.8)	33.5 (2.6)	≤ 28.87
**ROCF Recall** [35]	18.9 (16.4)	18.8 (6)	≤ 9.46
**Stroop Reaction Times** [36]	19.6 (8.3)	11.7 (7.5)	≥ 36.92
**Stroop Errors** [36]	1.2 (2.2)	0.5 (2.1)	≥ 4.24
**Phonemic Fluency** [37]	41.3 (10.7)	38.9 (11.2)	< 17.35
**Geriatric Depression Scale** [38]	5.1 (3.7)	/	> 14

Mean raw and correct scores (standard deviation in parentheses) at each neuropsychological test. Cutoff scores indicate the value above/below which the cognitive performance is considered pathological.

Abbreviations: MMSE = Mini Mental State Examination; RAVLT = Rey’s Auditory Verbal Learning Test; RCPM = Raven’s Coloured Progressive Matrices; TMT = Trail Making Test; ROCF = Rey-Osterrieth Complex Figure.

### Stimuli and procedure

Stimuli were colored and light grey dots (30 cd/m2, with a diameter of 1°), presented on a dark grey background (20 cd/m2). The colors used were: red (RGB [250, 0, 0]), blue (RGB [0, 20, 165]), yellow (RGB [250, 250, 0]), light green (RGB [0, 250, 0]) and purple (RGB [139, 58, 98]) for dots presented in the ‘relevant hemifield’ (targets), and orange (RGB [255, 127, 0]), light blue (RGB [64, 224, 208]), dark green (RGB [34, 139, 34]), pink (RGB [255, 105, 180]) and brown (RGB [139, 69, 19]) for dots presented in the ‘irrelevant hemifield’ (distracters). To (at least partially) exclude the effect of spatial proximity between targets and distracters, which may have a role in modulating vWM (e.g., [11, 12, 13]), we chose to present targets and distracters in separate hemifields (see also [24] in young adults only). In each trial either 1, 2 or 4 colored dots were independently presented in each side of the screen together with grey dots, resulting in the same or different number of colored dots across the two hemifields. In order to equate the sensory information presented on both sides, the total number of stimuli presented on the screen was kept constant throughout the experiment (18 items in total: 9 items for each hemifield, comprising colored + grey dots). The items were positioned using an invisible 8 (rows) by 10 (columns) (13.8° x 16.4°) grid centered at the center of the screen, where a white fixation cross was present for the entire trial procedure. Colored dots never appeared in the extreme rows and columns or in the columns closest to the fixation cross.

**Fig 1:**
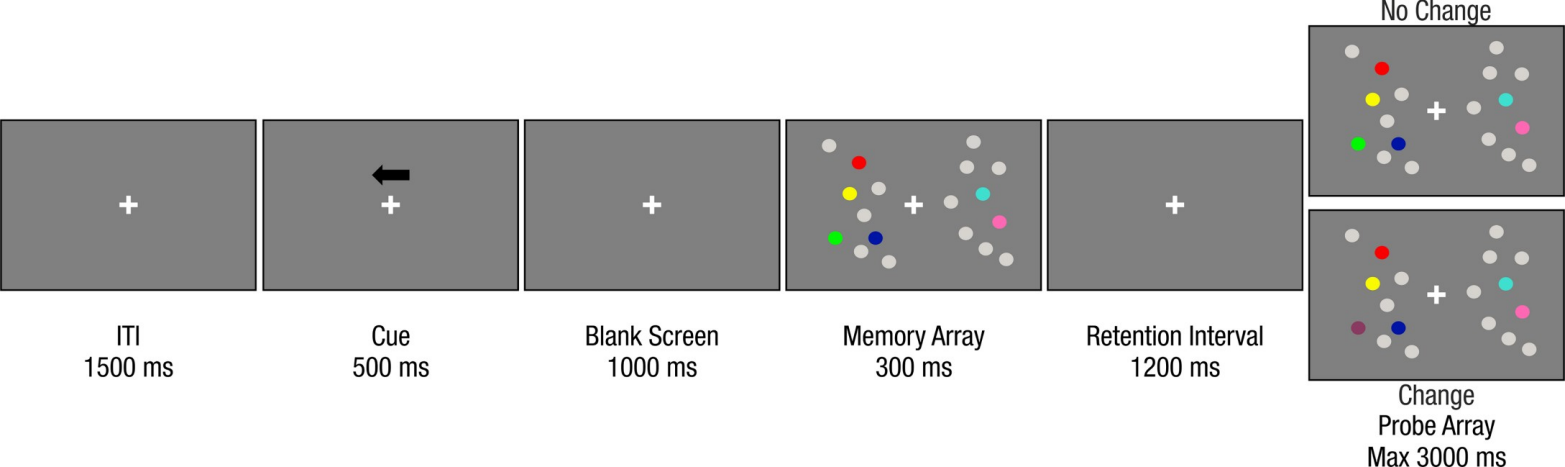
Stimulus sequence of a trial. An example of a condition where there is a numerical dissimilarity between targets (cued hemifield, 4 elements) and distracters (uncued hemifield, 2 elements).

Participants sat in front of a 19-inc LCD monitor (resolution 1280 x 1024, refresh rate of 75 Hz, viewing distance of 85 cm) and performed a change detection task on lateralized stimuli (Fig 1). In each trial, after a 1500 ms inter stimulus interval, a black arrow (3.3°) appeared for 500 ms above the central fixation cross. The arrow pointed randomly and with equal probability leftward or rightward, signaling the to-be attended hemifield (‘relevant hemifield’). The arrow cue was always valid. After 1 second, the memory array appeared for 300 ms, followed by a 1200 ms retention interval. Participants had to memorize the colors of the stimuli in the cued relevant hemifield (targets). On 50% of the trials, the test array was identical to the memory array (i.e. no change condition), while in the remaining 50% of the cases one target in the relevant hemifield changed color (i.e. change condition). Participants were informed that the colors of the distracters in the irrelevant hemifiled never changed. The test array remained on the screen until response, or for a maximum of 3 seconds. Participants reported whether the probe differed or not with respect to the memory array by pressing a key (letter M or C) on the keyboard. Response assignment to each key (‘same’, ‘different’) was counterbalanced between subjects. Participants completed a total of 720 trials divided in 15 blocks of 48 trials each, after performing a practice block of 10 trials. Each block comprised 24 trials where targets (relevant hemifield) and distracters (irrelevant hemifield) shared the same numerosity (8 trials for each shared numerosity: 1, 2, 4), and 24 trials where there was a numerical disparity between the two sides (4 trials for each possible numerosity combination of targets and distracters).

### EEG recordings and analysis

EEG was continuously recorded using 29 active electrodes placed according to the 10–20 International System (Fp1, Fp2, F7, F3, Fz, F4, F8, FC5, FCz, FC6, T7, T8, C3, Cz, C4, CP5, CP6, P7, P3, Pz, P4, P8, PO7, PO9, PO8, PO10, O1, Oz, O2), with a digitization rate of 1000 Hz, a time constant of 10 s as low cut-off and a high cut-off of 250 Hz. AFz served as ground and the right mastoid as the on-line reference. Horizontal ocular movements were recorded using two electrodes placed on the outer canthi of both eyes. Electrode impedance was kept below 20 kΩ.

The continuous EEG signal was processed off-line using EEGLAB [39] and ERPLab [40]. Data were down-sampled to 250 Hz and filtered with a low-frequency cutoff of 0.1 Hz and a high-frequency cutoff of 40 Hz. In order to remove the 50 Hz line noise, a notch band-pass filter (width: 2 Hz) was also applied. All channels were re-referenced to the average of the left and right mastoids. Independent component analysis (ICA) was applied to the whole dataset (Infomax ICA algorithm, [41]) to correct for eye blinks, muscle and cardiac activity. Epochs with correct responses were segmented from -200 ms to 1 second relative to the onset of the memory array, with a baseline correction of 200 ms pre-stimulus onset. Epochs were visually inspected and those contaminated by large eye movements or residual noise were removed. Finally, epochs were collapsed across change condition (change, no change) and target side (left, right), to obtain contralateral and ipsilateral activity regardless of the actual cue direction. A total of six different conditions were extracted (target load x target-distracter numerical similarity): Load1 –Same Numerosity (SN), Load1 –Dissimilar Numerosity (DN), Load2- SN, Load2 –DN, Load4 –SN and Load4 –DN. After pre-processing, the mean number of epochs retained for the average in the Young group was 95.16 for Load1 –SN, 93.68 for Load1 –DN, 94.13 for Load2 –SN, 93.52 for Load2 –DN, 82.94 for Load4 –SN and 85.61 for Load4 –DN. In the Old group the mean number of epochs retained for the average was 91.94 for Load1 –SN, 91.03 for Load1 –DN, 89.25 for Load2 –SN, 88.19 for Load2 –DN, 66.88 for Load4 –SN and 67.22 for Load4 –DN.

To investigate alpha-band lateralization changes, a time-frequency (TF) analysis was performed with a zero-padded complex Morlet wavelet decomposition of 5 cycles per frequency, as implemented in Fieldtrip toolbox [42]. Power was calculated for frequencies from 1 to 40 Hz (frequency resolution: 1 Hz) by sliding a time window over each trial in steps of 20 ms (from -2.5 to 2.5 s, relative to the memory array onset). Resulting TF data were averaged across correct trials collapsed for target side (see above for the six different load x similarity conditions and mean number of trials used) and then baseline corrected (-1.8 to -1.6 s with respect to memory array onset) in order to investigate relative changes in power (i.e. post-target power / baseline power).

### Statistical analysis

#### Behavioral data

For each subject and condition, the memory capacity index [43] was computed as follows: k = (hit rate–false alarm rate) * load. Load refers to the number of colored target dots that participants had to remember. Hit rates were defined as ‘different’ responses in change conditions, while false alarms were ‘different’ responses in no change trials. An analysis of variance (ANOVA) was conducted with Age (2 levels: young, old) as between-subjects factor, and Load (3 levels: 1, 2, 4) and Numerical Similarity (2 levels: same, dissimilar) as within-subjects factors. When significant, any interaction involving Load as a factor was further analyzed by considering only the two extreme values (i.e. 1 and 4 targets), in order to reduce the complexity of the analyses.

For both behavioral and electrophysiological data (see description below), in case of violation of sphericity, Greenhouse-Geisser (when G-G epsilon < .75) or Huynh-Feldt (when G-G epsilon >.75) correction was used, and adjusted *p* values are reported. All follow-up pairwise comparisons were conducted through t-tests. Correction for multiple comparisons was performed using the False Discovery Rate (FDR) procedure [44].

#### ERP data

To assess the temporal evolution of the electrophysiological correlates of active maintenance in vWM after the memory array onset, and following previous studies [11, 13], the ERP analysis was performed in two consecutive steps. First, a main temporal window of interest was analyzed by computing the lateralized activity (contralateral–ipsilateral activity with respect to the cued hemifield) for each condition in a region of interest (ROI) comprising electrodes O1/2, P7/8 and PO7/8 (see [45]) over an interval from 300 to 900 ms after the memory array onset (the typical time range used for the analysis on CDA, see [14]). An ANOVA was carried out on mean amplitude values, with Age as between-subjects factor, and Load and Numerical Similarity as within-subjects variables.

Second, significant main or interaction effects resulting from the main ANOVA were separately investigated (via paired-samples t-tests, and comparing 1 and 4 target-trials only for Load, see [12]) over consecutive time windows of 20 ms (see [11, 13] for a similar approach). A significant difference for at least 2 consecutive time windows (i.e. 40 ms) was considered reliable.

#### Alpha lateralization

To characterize the time course of alpha-band lateralization, relative power changes were averaged over alpha frequencies (8–14 Hz) in the whole retention interval window (from 300 to 900 ms after memory array onset, hereafter referred to as “post-target” onset). Mean relative power change values were computed for the two posterior contralateral- and ipsilateral-to-target ROIs (always including electrodes O1/2, P7/8, PO7/8). An ANOVA with Age as between-subjects and Hemisphere (2 levels: contralateral, ipsilateral), Load and Numerical similarity as within-subjects variables was performed to investigate relative power changes occurring during the retention interval.

## Results

### Behavioral

#### K (WM item capacity)

The ANOVA indicated a significant main effect of Group (F(1, 61) = 95.60, p < .001, η_p_^2^ = .610): young adults exhibited overall higher vWM capacity (M = 1.91, SD = .18, 95% CI = [1.84 1.97]) than older adults (M = 1.37, SD = .25, 95% CI = [1.28 1.46]). The effect of Load (F(2, 122) = 369.81, p < .001, η_p_^2^ = .858), of the interactions between Load and Group (F(2, 122) = 113.54, p < .001, η_p_^2^ = .651) and between Load and Numerical similarity (F(2, 122) = 4.08, p = .044, η_p_^2^ = .063) were also significant. The three-way Group x Load x Numerical similarity interaction was not significant (p = .213).

**Fig 2:**
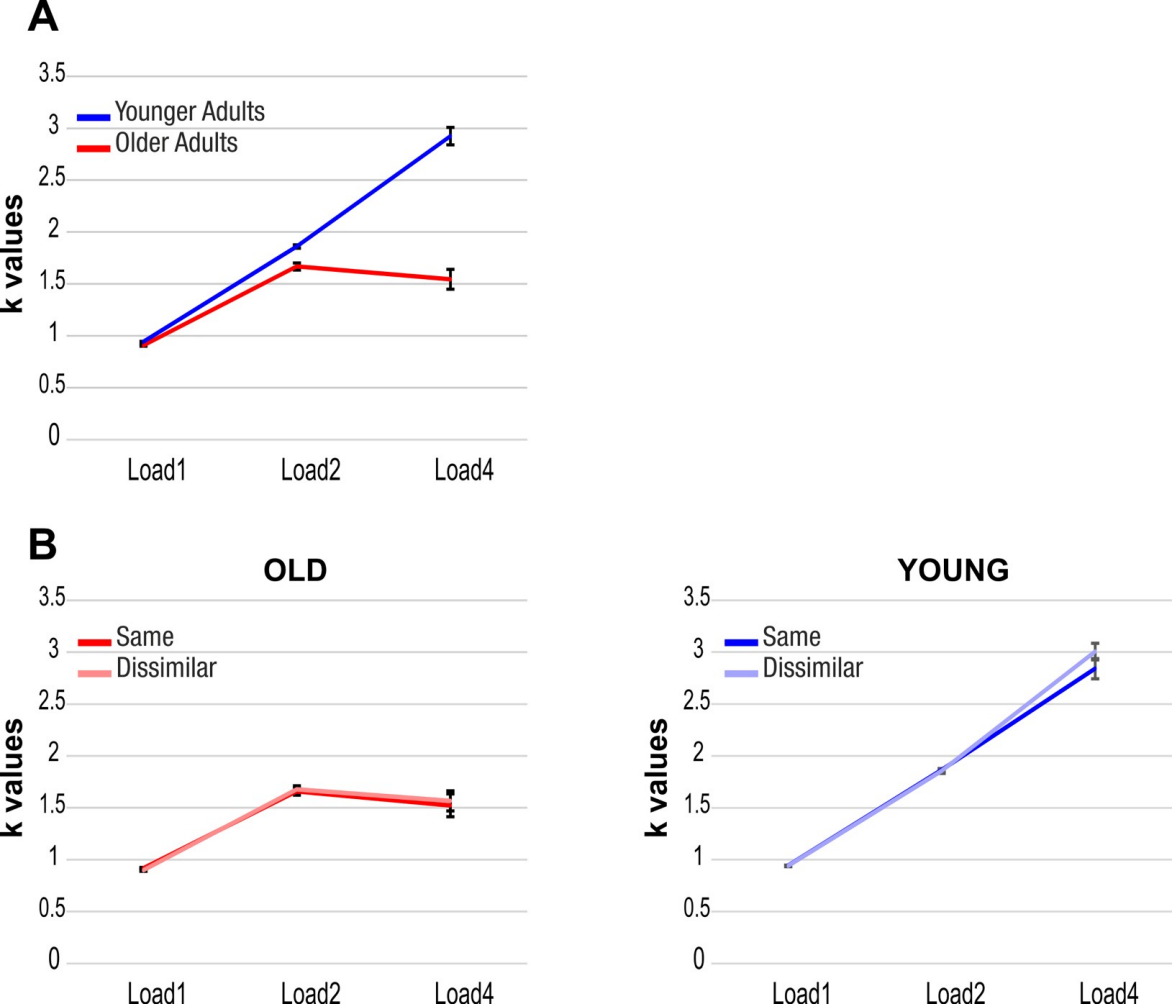
WM capacity. K values for young (blue line) and older (red line) adults (A) and for same and dissimilar numerosity in the two groups (B). Vertical bars represent standard errors.

Follow-up comparisons were conducted in two steps for the Load x Group interaction. First, in young adults the comparison between Load1 and Load4 indicated that vWM capacity increased with increasing target load (t(30) = -23.91, p < .001, 95% CI = [-2.15–1.81]) (Fig 2A, blue line). Also in older participants, post-hoc comparisons between Load1 and 4 revealed an increase in vWM capacity from Load1 to Load4 (t(31) = -6.86, p < .001, 95% CI = [-.83 -.45]) (Fig 2A, red line). Thus, vWM capacity of both groups increased with target load in both similarity conditions. Then, the difference between Load4 and Load1 (i.e. vWM increase) was computed for each group and compared between young and older participants. The comparison between the two groups in the vWM capacity increase revealed a significant difference (t(61) = -10.79, p < .001, 95% CI = [-1.60–1.10]), showing that the increase of k values was larger in the young (M = 1.98, SD = .46, 95% CI = [1.81 2.15]) compared to the older group (M = .64, SD = .53, 95% CI = [.45 .83]) (Fig 2A).

For the Load x Numerical similarity interaction, post-hoc comparisons were also performed in two steps. First, comparisons between Load1 and 4 in either similarity level indicated a significant difference (same numerosity: t(62) = -11.34, p < .001, 95% CI = [-1.46–1.02]; dissimilar numerosity: t(62) = -12.60, p < .001, 95% CI = [-1.57–1.14]), with higher WM capacity for Load4. Then, to compare the increase in memory capacity between the two numerical conditions as a function of memory load, difference k values (Load4—Load1) were again computed. A significant difference was found (t(62) = -2.20, p = .032, 95% CI = [-.22 -.01]), indicating a slightly larger increased WM capacity in the dissimilar than same numerical condition (Fig 2B).

#### Control analyses

In the dissimilar numerical condition, trials with 1 or 4 targets were always associated with either more (1 target) or fewer (4 targets) distracters. To evaluate the effect of numerical similarity in a more balanced condition (i.e., in trials where the number of distracters could be both smaller and larger than the target numerosity), we conducted two further analyses for two-target and two-distracter trials, respectively.

On the basis of the results of the main analysis on k values, two subsequent repeated-measures ANOVAs were performed to further explore the significant interaction between Load and Numerical similarity.

The first ANOVA was conducted on trials with 2 targets, with Distracter as a within-subjects variable (3 levels: 1, 2 and 4). The factor was not significant (p > .05), suggesting that the performance when 2 targets were presented was not modulated by the number of distracters in the irrelevant hemifield.

The second ANOVA was conducted on trials with 2 distracters, with Load as a within-subjects variable (3 levels: 1, 2 and 4). The factor was significant (F(2, 124) = 135.35, p < .001, η_p_^2^ = .686). The follow-up pairwise comparisons revealed a significant difference between Load1 and Load2 (t(62) = -42.94, p < .001, 95% CI = [-.89 -.81]) and between Load1 and Load4 (t(62) = -12.73, p < .001, 95% CI = [-1.55–1.13]), but not between Load2 and Load4 (p > .05). These results indicate that the limit of vWM capacity is between two and four targets.

Taken together, the behavioral analyses do indicate a genuine effect of numerical similarity on participants’ performance (but likely an effect driven by distracter numerosity at Load4; see Fig 2B).

### Event-related potentials (ERPs)

#### 300–900 ms (lateralized activity)

The results indicated the significance of Load (F(2, 122) = 57.84, p < .001, η_p_^2^ = .487), Numerical similarity (F(1, 61) = 5.61, p = .021, η_p_^2^ = .084) and of the interactions between Load and Group (F(2, 122) = 5.54, p = .005, η_p_^2^ = .083) and between Load, Numerical similarity and Group (F(2, 122) = 3.163, p = .046, η_p_^2^ = .049). To further explore the significant three-way interaction, we conducted a series of t-tests over 20 ms time windows comparing Load1 and Load4 in each numerical similarity condition and for each age group separately (see Methods for a detailed explanation).

**Fig 3:**
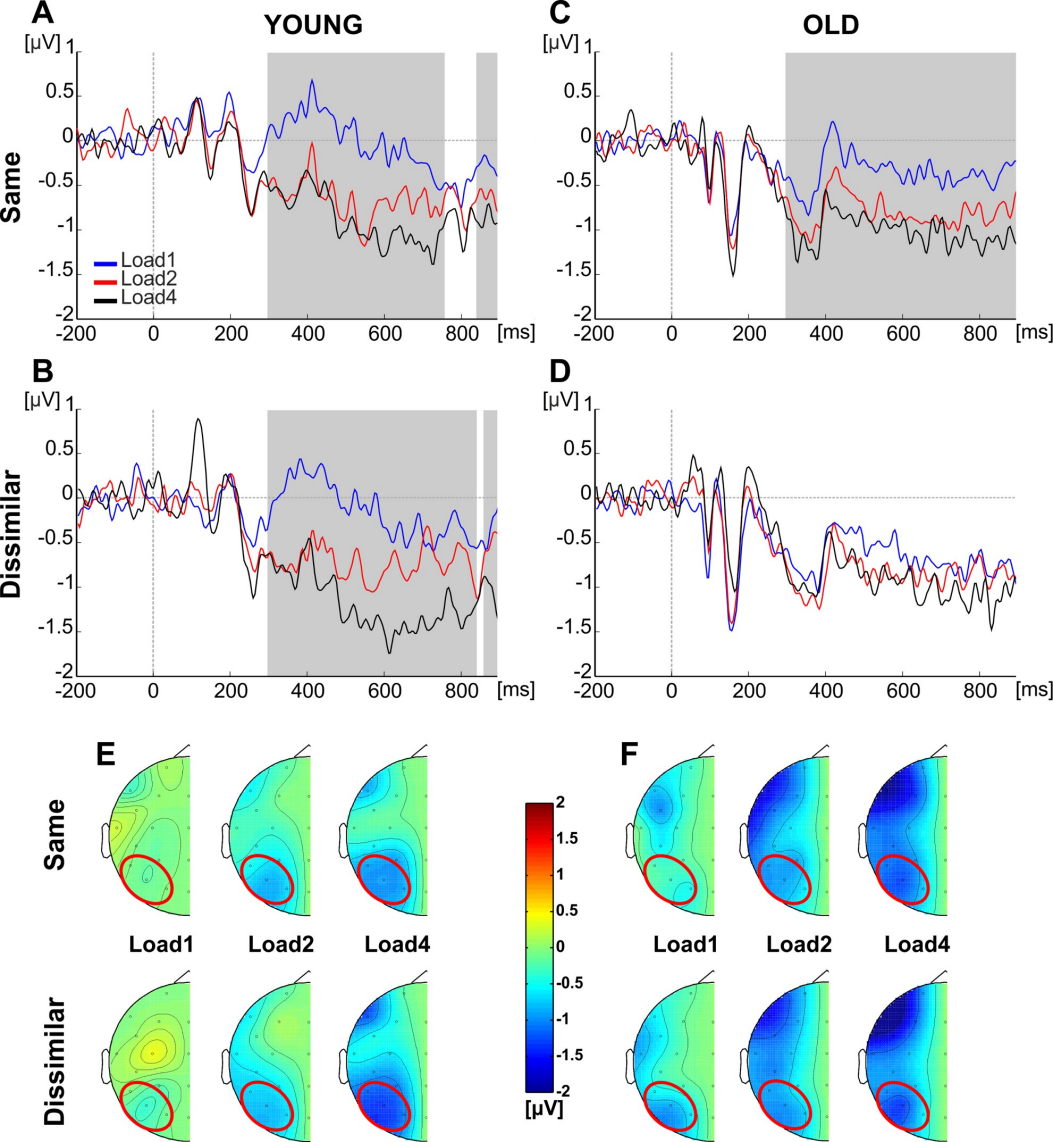
Contralateral delay activity. Grand average difference waveforms as a function of target load over the ROI (P7/8, PO7/8, O1/2). (A) Young–same numerosity. (B) Young–dissimilar numerosity. (C) Old–same numerosity. (D) Old–dissimilar numerosity. Grey squares indicate the significant time windows for the comparison between Load1 and Load4. (E, F) Topographical representations of the effects of load and similarity in the CDA time range in the Young (E) and Old (F) groups. Data were projected over one hemisphere only, as target side was collapsed. Red circles indicate the ROI considered to compute the CDA.

In young adults, in the same numerical condition a reliable difference between the two loads was evident from 300 to 740 ms and from 840 to 900 ms post memory array onset (all ps < .019; Fig 3A); similarly, in the dissimilar numerical condition significant differences emerged from 300 to 840 ms and from 860 to 900 ms (all ps < .034; Fig 3B).

In older adults, in the same numerical condition the difference between Load1 and Load4 was significant from 300 to 900 ms (all ps < .016; Fig 1C). Conversely, no significant difference was found for the dissimilar numerical condition between Load1 and Load4 (Fig 3D). Taken together, the results in older adults revealed that a modulation of the CDA as a function of target load was present only when the same number of targets and distracters were presented in the visual field.^1^

#### Control analyses

The same control analyses as for the behavioral data were performed on the CDA for trials with either 2 targets or 2 distracters. As the main analysis on mean amplitude values found a significant interaction between Load, Numerical similarity and Group, two subsequent mixed ANOVAs were conducted.

The ANOVA on 2-target trials, with Distracter as a within- and Group as a between-subjects variable, did not reveal any significant effect (all ps > .05), meaning that the CDA amplitude was not modulated by the number of distracters at Load2.

From the ANOVA on 2-distracter trials, with Load as a within- and Group as a between-subjects variable, a significant effect of Load (F(2, 122) = 21.63, p < .001, η_p_^2^ = .262) and of the interaction between Load and Group (F(2, 122) = 10.19, p < .001, η_p_^2^ = .143) emerged. Follow-up comparisons revealed a significant difference between Load1 and Load2 (t(30) = 3.77, p = .001, 95% CI = [.27 .91]), between Load2 and Load4 (t(30) = 4.76, p < .001, 95% CI = [.34 .84]) and between Load1 and Load4 (t(30) = 6.37, p < .001, 95% CI = [.80 1.55]) only in the group of young participants. No significant difference emerged in the older group (all ps > .05). Overall, when two distracters were presented, the amplitude of the CDA became more negative as a function of load only for young subjects.

Overall, the results indicated a CDA modulation as a function of target load for young participants regardless of numerical similarity. In older participants, there was an effect of target load on CDA only in the same numerosity condition; however, there was no CDA modulation of numerical similarity per se, as revealed by the control analyses.

### Alpha event-related synchronization/desynchronization (ERS/ERD)

#### Post-target interval

The mixed ANOVA showed a significant main effect of Load (F(2, 122) = 7.61, p = .001, η_p_^2^ = .111) and significant interactions between Hemisphere and Group (F(1, 61) = 8.99, p = .004, η_p_^2^ = .128), between Load and Group (F(2, 122) = 3.54, p = .032, η_p_^2^ = .055) and between Load and Hemisphere (F(2, 122) = 7.30, p = .001, η_p_^2^ = .107). The Load x Hemisphere interaction was not further investigated as we were mainly interested in age and numerical similarity effects.

**Fig 4:**
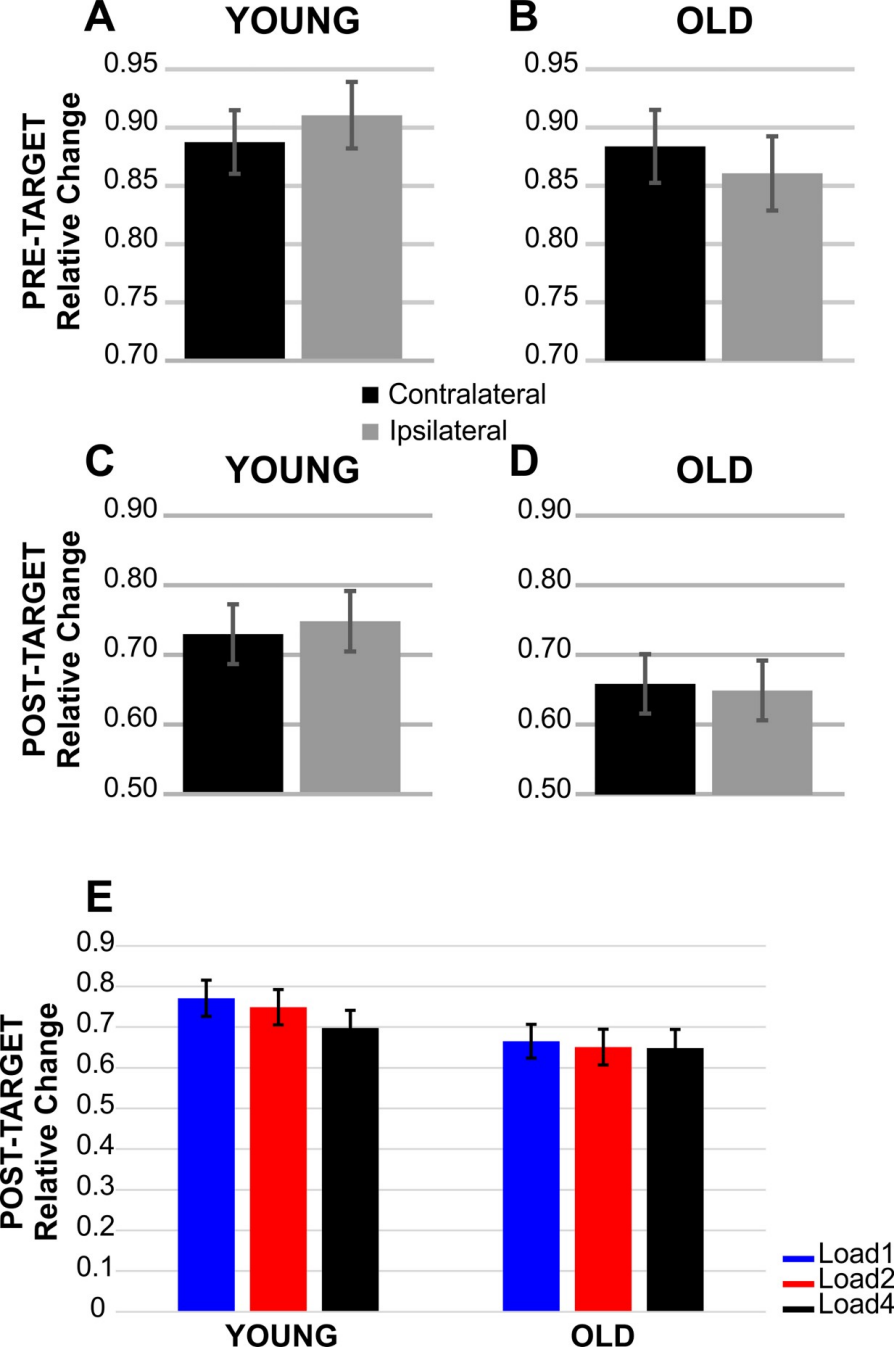
Alpha event-related synchronization/desynchronization. Relative alpha power changes over the pre-target (-200 to 0 ms) interval in (A) Young and (B) Old. Relative post-target (300 to 900 ms) alpha power changes in (C) Young and (D) Old. (E) Post-target global alpha modulation as a function of target load in the two groups. Vertical bars represent standard errors.

Given that alpha lateralization is measured as a power reduction for contralateral relative to ipsilateral sites [46], comparisons were conducted by means of one-tailed t-tests, separately for young and older adults. The pairwise comparisons revealed a lateralization effect in the young age group (t(30) = -2.26, p = .016, 95% CI = [-.04 -.002]), with the contralateral sites exhibiting greater alpha reduction than the ipsilateral ones (Fig 4C). In older adults, the trend of the lateralization went in the direction opposite to what expected (the ipsilateral was more negative than the contralateral hemisphere), hence the null hypothesis must be accepted (i.e, no significant difference between the two hemispheres; t(31) = 2.08, p > .05, 95% CI = [.0002 .02]) (Fig 4D).

To investigate the Load x Group interaction, in the young age group pairwise post-hoc comparisons between Load1 and 4 indexed greater alpha power decrease at Load4 than at Load1 (t(30) = 4.59, p < .001, 95% CI = [.04 .11]) (Fig 4E, left histogram). In the elderly, no significant difference emerged (p > .05) (Fig 4E, right histogram). Overall, a reduction in alpha power with target load was evident in the young but not in the older group.

In sum, in young participants the results showed a global alpha power suppression (i.e. irrespective of hemisphere) that covaried with memory load, thus confirming its role as an index of spatially global vWM representations [25, 47]. No such effect was visible for older participants. Moreover, alpha power lateralization favoring the target hemisphere was absent in the older group.

### Additional analyses

#### ERPs: 0–300 ms (lateralized activity)

Another temporal window of interest was analyzed by computing the lateralized activity in the ROI comprising electrodes O1/2, P7/8 and PO7/8, over a 0–300 ms interval after the memory array onset. This time window was included to control for possible differences between the two groups in the early stages of stimulus processing. An ANOVA was carried out on mean amplitude values, with Age as between-subjects factor, and Load and Numerical Similarity as within-subjects variables.

A significant main effect of Group emerged (F(1, 61) = 7.12, p = .010, η_p_^2^ = .105), showing that the older group exhibited more negative values (M = -.29, SD = .32, 95% CI = [-.40 -.17]) than the young (M = -.08, SD = .31, 95% CI = [-.19 .04]). Also a significant interaction between Load and Similarity (F(2, 122) = 4.93, p = .009, η_p_^2^ = .075) emerged. However, no reliable difference between the two numerical conditions emerged from the post-hoc analysis (multiple t-tests over consecutive 20 ms time windows).

Although we were mainly interested in the late time range (which is the typical latency range of the CDA), and despite the presence of significant main and interaction effects in our 0–300 ms analysis, we acknowledge that using such a large window for the earlier analysis could have in principle reduced the chance to find significant effects.

#### ERPs: 300–900 ms (lateralized activity over frontal areas)

Visual inspection of the topographical representations suggested an additional effect on a frontal cluster of electrodes (F3/4, F7/8 and FC5/6) in the CDA time range. Thus, we performed the same analyses as for the posterior ROI. The mixed ANOVA (factors: Group, Load and Numerical similarity) on the mean amplitude (300–900 ms time window) revealed significant main effects of Group (F(1, 61) = 5.22, p = .026, η_p_^2^ = .079), showing that elderly had more negative values (M = -1.15, SD = 2.06, 95% CI = [-1.89 -.41]) than the young (M = -.27, SD = .55, 95% CI = [-.48 -.07]), and Load (F(2, 122) = 19.59, p < .001, η_p_^2^ = .243). To further explore the Load effect, we conducted t-tests over 20 ms time windows comparing Load1 and Load4. Significant differences emerged from 320 to 900 ms (all ps < .046). To our knowledge, only [12] and [48] investigated lateralized ERPs in vWM by looking also at more anterior regions. Specifically, Sander and colleagues [12] found a significant effect in a similar region only in children and older participants, suggesting that it might reflect a greater engagement of prefrontal control processes. In a paradigm where distracters appeared together with targets in the relevant hemifield, Liesefeld et al. [48] instead revealed greater prefrontal activation in distracter-present conditions. In our experimental design, target elements were additionally embedded with non-salient items (grey dots) in the relevant hemifield, thus (partly) requiring more effort to perform the task. This might be the reason why the frontal effect was evident also in young participants (note also that single-neuron activity recordings in the primate identified a sustained activity in the prefrontal cortex as one of the physiological correlates of WM, see for example [49]). Moreover, the greater frontal activation observed in the older group is in line with the notion of a posterior to anterior shift in aging (PASA; [50]), with frontal regions compensating for the reduced activation of posterior areas. Overall, since the majority of the ERP studies on vWM and concurrent age-related decline have not investigated anterior regions, the functional significance of this effect deserves further investigation.

#### ERS/ERD: Pre-target interval

Following the results found for alpha lateralization during the post-target interval, we investigated ERS/ERD during the pre-target (i.e. post-cue) time window. TF data were averaged across all correct trials collapsed for cue direction and then baseline corrected (-1.8 to -1.6 s with respect to memory array onset) to measure relative changes in power. The mean number of trials used was 520.96 (72.36% of the total number of trials).

Relative power changes were averaged over alpha frequencies (8–14 Hz) in the last 200 ms preceding the memory array onset (see [51]), when the spatial bias induced by the cue (namely, a reduction in power for the contralateral sites relative to the ipsilateral sites) is supposed to be stronger [52]. Mean relative power change values were computed for the two posterior contralateral- and ipsilateral-to-cue direction ROIs (O1/2, P7/8, PO7/8). An ANOVA with Age as between-subjects and Hemisphere as within-subjects factors was conducted.

A significant interaction between Hemisphere and Group (F(1, 61) = 9.12, p = .004, η_p_^2^ = .130) was evident. The pairwise comparisons performed separately in each group through one-tailed t-tests revealed a lateralization effect in the young age group (i.e. greater alpha reduction in the contralateral than in the ipsilateral-to-cue-direction hemisphere; t(30) = -1.91, p = .033, 95% CI = [-.048 .002]) (Fig 4A). In the elderly, no difference between the two hemispheres was evident (t(31) = 2.43, p > .05, 95% CI = [-.001 .04]), as again the results went against predictions (the ipsilateral alpha power was more negative than the contralateral alpha power) (Fig 4B).

Overall, in line with the results on alpha lateralization in the post-memory array onset, young but not older participants exhibited greater cortical facilitation for the cued hemisphere.

## Discussion

In many everyday scenarios, individuals experience the need to act on multiple relevant objects that are presented amidst other irrelevant items sharing the same attributes, such as shape, color or numerosity. This type of similarity between targets and distracters can be a potential source of distraction, especially in senescence. The present study provides new information on 1) the effect exerted by numerical similarity on vWM in young and older adults and 2) how age-related distractibility modulates vWM capacity.

As expected [7], the behavioral results highlighted a reduction in performance for the group of older participants. Whereas the estimated number of elements retained (provided by k values) increased with target load in both groups, the increasing rate was larger for young adults (who could efficiently retain up to approximately three elements, while older participants reached their WM capacity limit at around two targets).

Numerical similarity seemed to slightly influence the performance of both young and older participants: k values were higher when targets and distracters had different numerosities, although the effect was not magnified by aging. Crucially, the similarity effect was not confirmed by the additional analysis investigating the influence of the number of distracters when subjects had to retain two target elements: following these comparisons, no behavioral advantage for the two dissimilar conditions (one and four distracters, respectively) emerged. By looking at the graph (Fig 2), it seems plausible that the interaction found in the main analysis is driven primarily by the difference between the same and dissimilar numerical conditions at the highest memory load, i.e. four targets. However, the presence of the effect only at Load4 could be explained by the disproportion between the numerosity of targets (four elements) and of distracters (always fewer than four) in this condition. Thus, the effect is likely driven by distracter numerosity rather than numerical similarity per se.

At the electrophysiological level, the CDA pattern associated with the distracter numerical similarity was crucial in unravelling two novel findings.

First, numerical similarity did not influence the load-related modulation of the CDA amplitude in young adults: the same modulation as a function of memory load was observed in both conditions (in line with [25]), and no significant effects of numerical similarity could be inferred from the control analyses. The effect of memory load was not persistent for the whole CDA interval, as the modulation ceased and then reappeared shortly before the probe onset. This result suggests that before the presentation of the probe array (always occurring at a fixed time interval after the target display onset) young participants refreshed the items in their WM buffer.

Second, in older participants the results of the main analysis showed an effect of numerical similarity, with a modulation of the CDA as a function of target load in the same but not in the dissimilar condition. Does this pattern imply that numerical similarity facilitated older adults in the memorization of targets when they have the same numerosity of distracters? On the basis of previous literature [14], a larger CDA modulation as a function of target numerosity indicates a better ability to maintain the relevant elements in vWM. However, on the basis of previous research [1, 2, 3, 4, 5, 6], in the present study the larger modulation should have been expected for the dissimilar (not the same) numerical condition. Therefore, the opposite pattern found for the modulation of the CDA observed here recommends caution with this interpretation.

**Fig 5:**
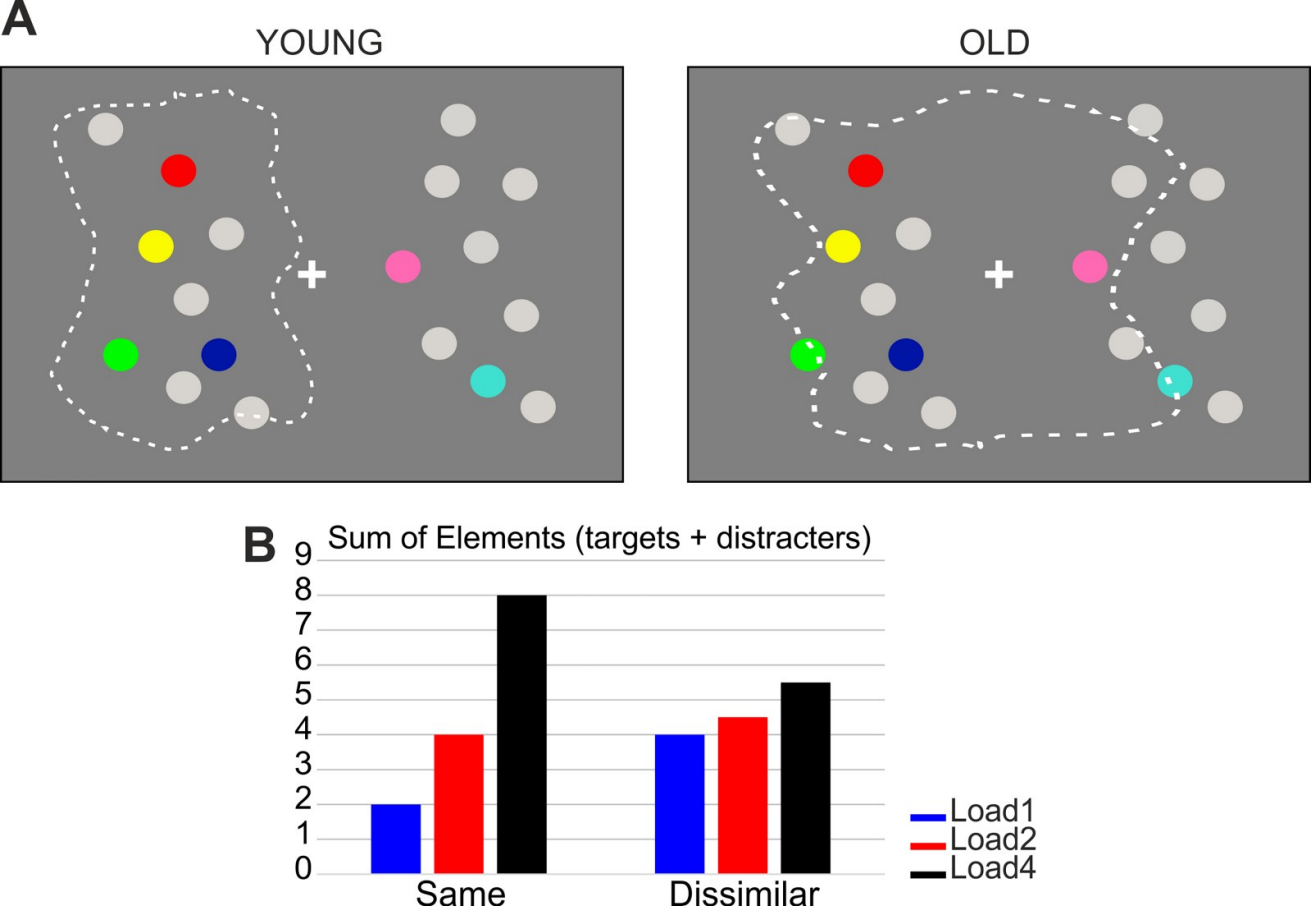
Enlargement of the processing field. (A) Graphic representation of the “age-related enlargement of memorization” field. The memorization field is limited to the relevant hemifield in young adults (left panel), but encompasses also part of the irrelevant hemifield in older adults (right panel). (B) Mean of the total number of elements (targets + distracters) across target load for the same numerical condition (where the number of targets and distracters is positively correlated, left histogram), and for the dissimilar numerical condition (where the number of targets and distracters is negatively correlated, right histogram). The number of overall elements in the dissimilar numerical condition results from an average of the number of elements of the two conditions collapsed at each memory load (e.g. Load2: 4.5 elements = [(2 targets + 1 distracter) + (2 targets + 4 distracters)]/2).

Alternatively, we could reconsider the entire profile of the EEG responses for older adults in terms of a substantial overlap in the analysis of the relevant and irrelevant hemifields, due to an age-related broadening of the processing field for the relevant side (Fig 5A).

According to previous research, in tasks engaging different cognitive abilities, including working memory, activity in several brain areas appears less lateralized in the elderly [53]. This reduced lateralization is thought to reflect either a compensatory function or a de-differentiation process. Moreover, models of deployment of spatial attention [54, 55, 56] predicted and proved that the focus of visuospatial attention becomes broader and less concentrated in healthy aging.

In line with these findings, we propose that older adults exhibit a weaker ability to focus processing resources towards a spatially delimited portion of the visual field, where relevant elements are expected or presented. As a consequence, they also tend to encompass a variable portion of the irrelevant visual field at various stages of analysis, ultimately achieving a less efficient behavioral performance with respect to young individuals. Therefore, we propose that in the memory retention phase (CDA), the different pattern related to target load for the same and dissimilar condition reflects the covariance between target and distracter numerosity in the current experimental design. In fact, in the same numerical condition, the number of targets and distracters was equal in each trial, so that the overall amount of elements presented in the visual field increased across target load (Fig 5B, left panel). Given the hypothesis of an age-related broadening of the processing field beyond the relevant side, one should predict that the memorization field encompassed (part of) the irrelevant side. However, the positive correlation in numerosity between targets and distracters in the same numerical condition ensures an overall increase in the number of (target and distracter) items retained (up until the limit of the WM capacity of the elderly, i.e. approximately 2 elements), as visible from the modulation of the CDA as a function of load in this specific condition.

Conversely, in the dissimilar numerical condition target and distracter numerosities were negatively correlated (i.e. when targets increased, distracters on average decreased, and vice versa), so that the global amount of elements presented on the screen was on average the same (i.e. around four) across all target loads (Fig 5B, right panel). Hence, due to the broadening of the “memorization field”, the number of items retained does not change across loads. Indeed, here the target load effect on the CDA disappears, given that the sum of all the elements always exceeds the WM limit of the elderly (i.e. the minimum amount of overall elements presented in the dissimilar numerosity conditions is three). The control analyses conducted on trials with two distracters seem to support this hypothesis, given that the CDA was modulated by target load in young but not older participants. Here again the minimum amount of overall elements is three (two distracters plus at least one target), which in turn exceeds the WM limit of the elderly.

The pattern of oscillatory data found in the present study supports the hypothesis of an age-related broadening of the processing field in the elderly. First, in line with previous results [57], the attention-related cortical facilitation induced by the cue was present in young participants but absent in the elderly, as revealed by the data on alpha lateralization after cue presentation. This pattern indicates that older adults tend to lose cortical facilitation for the relevant side, and therefore deploy attentional resources to both hemifields. Moreover, the same pattern of alpha lateralization persisted during the retention interval: lateralized alpha favoring the contralateral hemisphere was still present for young participants, while it was absent in the elderly (as in [28]). In fact, there was a trend towards an inversion of the alpha lateralization for the older group (with more negative values for ipsilateral than contralateral sites in both pre- and post-array intervals). While future studies replicating this observation are needed, we speculate that together with the overall pattern of alpha activity along the entire time window, this inversion supports our interpretation of the broadening of the memorization field. In addition, such interpretation entails that distracters should produce more interference when presented in a more medial/nasal than lateral/temporal position, a testable prediction for further research. According to the proposal that alpha lateralization as an index of suppression of irrelevant items [26], older adults did not show an enhancement of the relevant hemifield (i.e. lack of alpha lateralization) and processed also the distracting material presented in the irrelevant side (i.e. no distracter suppression). However, we prefer to remain agnostic as to the specific functional role of alpha lateralization, and to report the absence of lateralization as revealing an age-related broader focusing of processing resources.

Finally, the overall increase of the amplitude of the early lateralized ERP activity (0–300 ms window post-target onset) in older with respect to young participants seems to indicate a delayed attempt made by older participants to tune their processing resources exclusively towards the relevant hemifield (see [58]), although this was not sufficient to completely prevent distracters from being memorized (as reflected by the CDA load-related pattern).

Two aspects about this study should be considered. First, the majority of older participants performed at ceiling (i.e. obtaining an equivalent score of four) in the neuropsychological tests administered, thus showing a high level of cognitive functioning. It would be interesting in future research to investigate a sample of older individuals with higher variability in cognitive functioning. One could speculate that distractibility would increase in healthy elderly with a lower cognitive profile. Second, since the task was performed on a computer, we cannot totally rule out the impact of expertise with technological devices on the difference in performance between young and older adults. However, since participants were only required to provide responses by pressing one of two keys over a relatively long time period, computer expertise should have only minimally contributed to the present results.

To conclude, the behavioral and EEG pattern indicates that young adults do not suffer from distraction due to numerical similarity. In older participants, the effect of numerical similarity on the CDA was instrumental to get an insight on the nature of distractibility in the elderly. We propose that age-related fluctuations in endogenous attention, when coupled with the simultaneous presentation of targets and distracters in opposite hemifields, may result in a redistribution of the vWM resources across the two visual fields. This resource-consuming enlargement of the “memorization” field in turn affects the vWM capacity of older adults, and their performance compared to younger individuals.

## Supporting information

S1 File K values. Dataset containing the k values (Sheet1: k values of each Load * Numerical Similarity condition; Sheet2: k values of each Load2 * Distracter numerosity condition; Sheet3: k values of each Load * Distracter2 condition).
(XLSX)

S2 File CDA amplitude. Dataset containing the mean amplitude computed in the CDA time range (Sheet1: CDA of each Load * Numerical Similarity condition; Sheet2: CDA of each Load2 * Distracter numerosity condition; Sheet3: CDA of each Load * Distracter2 condition).
(XLSX)

S3 File ERP time course. Dataset containing the mean amplitude computed in 20 ms time windows from 0 to 900 ms after stimulus onset (Sheet1: Load1 –Same Numerosity; Sheet2: Load1 –Dissimilar Numerosity; Sheet3: Load4 –Same Numerosity; Sheet4: Load4 –Dissimilar Numerosity).
(XLSX)

S4 File Alpha power. Dataset containing the mean alpha power (Sheet1: Alpha power over post-cue interval for each hemisphere; Sheet2: Alpha power over post-target interval for each Load * Numerical Similarity * Hemisphere condition).
(XLSX)
